# Affordable SARS-CoV-2 protein vaccines for the pandemic endgame

**DOI:** 10.1038/s41541-022-00507-8

**Published:** 2022-08-02

**Authors:** James A. Triccas, Joeri Kint, Florian M. Wurm

**Affiliations:** 1 School of Medical Sciences, Faculty of Medicine and Health, The University of Sydney, Camperdown, NSW Australia; 2 Sydney Institute for Infectious Diseases and the Charles Perkins Centre, The University of Sydney, Camperdown, NSW Australia; 3 ExcellGene SA, 1870 Monthey, Switzerland; 4 Faculty of Life Sciences, Swiss Federal Institute of Technology Lausanne (EPFL), Lausanne, Switzerland

**Keywords:** Protein vaccines, Recombinant vaccine

The astonishing speed with which coronavirus disease 2019 (COVID-19) vaccines were developed is nothing short of a scientific triumph. Their rapid development was in part enabled by the use of novel gene-delivery technologies that could be manufactured quickly and at scale^1^. Both the mRNA (e.g. BNT162b2, mRNA-1273) and adenovirus-based vaccines (e.g. ChAdOx-1 nCov-19, Ad26.COV2.S) deliver genetic material to cells to instruct the production of the SARS-CoV-2 spike protein. These vaccines have saved many lives, with estimates of vaccination averting at least 1 million deaths within the USA alone^2^. While the use of these new vaccine technologies in high-income countries (HICs) has dramatically reduced the number of COVID-19-related hospitalisations and deaths, vaccination rates in low- and middle-income countries (LMICs) remain concerningly low. For example, as of May 8, 2022, only 17% of the eligible population in Africa have received two vaccine doses^3^. Vaccine coverage in LMICs has relied on ‘traditional’ technology approaches, namely inactivated viral vaccines. The CoronaVac (Sinovac), BBIBP-CorV (Sinopharm) and COVAXIN (Bharat Biotech) comprise around half of all vaccines delivered globally and have played an important role in controlling COVID-19 in LMICs^4^. Unlike mRNA vaccines, inactivated vaccines do not require long-term storage at very low temperatures, thus facilitating their use in LMICs. However, a limitation of these vaccines is the reduced capacity to neutralise infection with the Omicron variant, particularly in the absence of any additional booster dose after the initial vaccine course^5^. Disparities in vaccine access further compound this problem. Administration of booster doses in HICs has been prioritised over equitable vaccine distribution, thereby impacting effective control of global COVID-19 infections, deaths and emergence of new variants. Mechanisms to distribute vaccines to LMICs have also faced serious problems. The COVID-19 Vaccines Global Access Scheme (COVAX) was established to facilitate global COVID-19 vaccine distribution; however, COVAX is underfunded, has struggled to secure enough vaccine doses and has failed to meet many key targets. It is clear that global vaccine production and supply need to be increased, ideally through the building of vaccine manufacturing and distribution capacity in LMICs.

A traditional approach to developing vaccines against pathogens is the combination of antigenic components with immune-stimulating adjuvants. These protein-subunit vaccines have been in use for decades and have an excellent record in terms of safety and efficacy^6^. Because of their lack of complexity, these vaccines can be manufactured cost-effectively in commonly available biotechnology facilities. Like mRNA vaccines, protein vaccines can also be rapidly deployed to target new viral variants^7^. In addition, protein subunit vaccines can be stored and transported at ambient temperature and can induce long-lasting immunity^6^. The Novavax NVX-CoV2373 nanoparticle-based vaccine demonstrated a low rate of adverse events with protective efficacy equivalent to the Pfizer and Moderna mRNA vaccines^8^. As a consequence, this class of vaccines will be an important tool in the continuing battle to control COVID-19.

To enable true worldwide and equitable access to COVID-19 vaccines, governments and non-governmental organisations should focus investment on the clinical development of subunit vaccine candidates that can be mass-manufactured using widely available and well-established technologies. The most obvious production platform for such vaccines are Chinese hamster ovary (CHO) cells, producing the vast majority of therapeutic proteins in clinical use, some in the multi-ton range^9^. Using these systems, viral proteins, including SARS-CoV-2 trimeric spike antigens^10,11^, could be mass-produced at scale for a low cost. When combined with an affordable adjuvant, a sub-dollar per dose COVID-19 vaccine is feasible; this contrasts with the high cost of mRNA vaccines and complexities around their manufacture and access to intellectual property^12^. Further, rapid advances in adjuvant technology allow the specific tailoring of immune responses to the antigen^13^; this property has been exploited more broadly with the development of inactivated vaccines, as evidenced by the use of the Th1-promoting Alhydroxiquim-II adjuvant as part of the COVAXIN COVID-19 vaccine^14^. Protein subunit vaccines may serve as the best option for both primary vaccination series and heterologous boosting of previous vaccination, considering the unknown impact of multiple mRNA dosing, and the issue of pre-existing adenovirus vector immunity^15^. The use of protein subunit vaccines incorporating adjuvants such as alum (or derivatives) may facilitate sustained immunity and thus could overcome the waning of immunity observed with mRNA vaccines^16^. To further ensure equitable access, such vaccines could be mass-manufactured on different continents. Accordingly, a focus should be on enabling technology transfer and capacity building for regional vaccine manufacture and distribution in LMICs. We believe this truly global approach is required to stem the current COVID-19 pandemic, as well as future pandemics.
